# An increase in flow-diverter oversizing values as an independent risk factor for developing more severe in-stent stenosis. A retrospective single-center study based on flow diversion of supraclinoid internal carotid artery aneurysms

**DOI:** 10.3389/fneur.2024.1499732

**Published:** 2025-01-08

**Authors:** Georgi Vladev, Alexander Sirakov, Svetozar Matanov, Kristina Sirakova, Kristian Ninov, Stanimir Sirakov

**Affiliations:** ^1^Department of Interventional Radiology, University Hospital St. Ivan Rilski, Sofia, Bulgaria; ^2^Radiology Department, Medical University of Sofia, Sofia, Bulgaria

**Keywords:** DAPT, dual antiplatelet therapy, DSA, digital subtraction angiography, aneurysm, in-stent stenosis, embolization

## Abstract

**Introduction:**

In the past decade, flow diverters (FDs) have increasingly been used to treat cerebral aneurysms with unfavorable morphology in which other endovascular techniques fall short of being as effective. In-stent stenosis (ISS) is one of the most puzzling and frequent risks of flow diversion therapy observed on follow-ups. This complication, although mostly placid in its clinical course, can have dire consequences if patients become symptomatic. ISS is associated with many factors, none of which have been demonstrated to date to be solely responsible for the phenomenon.

**Methods:**

This study was aimed at evaluating ISS incidence in patients in our clinic who were treated with flow-diverters for aneurysms, located on the supraclinoid segments of the internal carotid artery between September 2022 and May 2023. A retrospective analysis was conducted, which included 137 patients with a total of 142 aneurysms being treated. The main hypothesis was that oversizing of the implant might play a role in ISS development. The performed statistical analysis, aimed at finding a correlation between it and vessel lumen narrowing on the follow-ups. The effects of other known risk factors, such as sex, age, smoking, and hypertension, were also analyzed.

**Results:**

Stent oversizing with respect to the parent artery was positively correlated with subsequent ISS occurrence and severity. Older age was a protective factor against ISS. Patients who actively smoked had diminished risk of developing severe ISS.

**Discussion:**

Stent oversizing can lead to ISS development, which might be more pronounced with larger implant-to-vessel sizing discrepancies. To achieve optimal results, the choice of implant diameter should consider all segments of the vessel in which it will be implanted. In cases of severe symptomatic ISS, continuation of dual anti-platelet therapy is a reasonable and effective option to address this complication.

## Introduction

In the treatment of cerebral aneurysms, flow diversion method has become frequently used by neurointerventionalists (1). This method has notably high efficacy and is a favorable option for treating complex wide-necked aneurysms (2). Its therapeutic window has been expanded to include smaller aneurysms with unfavorable morphology, which carry a high risk of coil embolization because of the potential for coil herniation into the parent vessel. Flow diversion is distinguished by high occlusion rates and the lack of a requirement for intrasaccular navigation, as is necessary with simple and adjunctive coiling techniques (3, 4). A drawback to the use of these intracranial stents is the need for continuous dual antiplatelet therapy (DAPT), to avoid implant thrombosis and associated ischemic complications (5). Another potential drawback is the potential for covered side-branch occlusion. Although the clinical significance of this occurrence remains debated, treating physicians should consider this aspect during decision-making and avoid it if possible (6).

The intra- and periprocedural complications of flow diversion can be divided into two main categories: ischemic and hemorrhagic (7). Hemorrhagic complications can occur both during embolization, owing to vessel injury and aneurysm rupture, and post-procedurally, as in delayed aneurysm ruptures, owing to aneurysm wall lysis after rapid thrombosis inside the sac (8, 9). Ischemic complications have been reported in approximately 7–10% of patients receiving flow diversion (10, 11). One potential late ischemic complication is the development of in-stent stenosis (ISS) after device implantation. Despite the effectiveness of prescribed antithrombotic medications, ISS is encountered in 10–50% of flow diversion cases (12, 13). ISS development, although frequently asymptomatic, can potentially lead to irremediable neurological damage.

Although several hypotheses regarding ISS and its formation have been proposed, the essence and exact pathophysiology of this condition largely remain unclear (14). Factors intrinsic to the implant and/or the treated vessel play crucial roles in ISS development. The consensus regarding ISS pathophysiology is that device implantation causes vascular injury, thereby initiating a biochemical inflammatory cascade (15). Platelet aggregation and subsequent leukocyte infiltration are the first events in response to this injury. Gradually, the secretion of various growth factors and cytokines leads to the activation and proliferation of the vessel endothelium, which begins to cover the struts of the stent (16). Vascular smooth muscle cells are also a vital part of the process because their activation due to induced stress from the stent can lead to vessel remodeling in later phases (17, 18). An additional pathological mechanism arising from endothelial cell damage is endothelial nitrous oxide synthase dysfunction, which plays crucial roles in multiple pathways associated with thrombosis and adhesion molecule expression (19). Other factors known to affect endothelial physiology and ISS development include age, sex, hypertension, and smoking (20–22).

Herein, this study sought to establish whether a correlation between stent oversizing and ISS development might exist in patients with supraclinoid internal carotid artery (ICA) aneurysms treated with flow diversion. Other previously described risk factors, such as age, sex, hypertension, and smoking status, were also examined to assess their effects on ISS development (23).

## Materials and methods

A retrospective study of patients with internal carotid aneurysms treated in our hospital between September 2022 and May 2023 was conducted.

### Inclusion criteria

1. At least one treated supraclinoid (C6 or C7 segment) ICA aneurysm treated with placement of a flow diverter stent.
2. Adequate compliance with the prescribed dual antiplatelet therapy, consisting of 10 mg prasugrel and 100 mg aspirin daily for at least 6 months.
3. Follow-up conventional angiography or magnetic resonance imaging (MRI) performed after treatment.
4. No immediate adverse effects or clinical worsening was observed after the procedure.

### Exclusion criteria

1. Age younger than 18 years.
2. Treatment in the setting of a subarachnoid hemorrhage.
3. Known allergies to platinum, nickel, and/or titanium.
4. Pregnancy, breastfeeding, or planned pregnancy in the next 12 months.

### Procedural aspects

Before treatment, all patients were prescribed DAPT, consisting of 10 mg prasugrel and 100 mg aspirin once daily for 5 days.

All endovascular procedures were performed on the department’s biplane angiographic system (Philips Azurion neuro 7 B20/15) under general anesthesia. In all cases, the vascular access was the right femoral artery *via* a 6-F introducer sheath. The general protocol involved four-vessel panangiography, after which a guiding catheter was placed in the carotid artery of interest. Navigation with a microwire and a microcatheter was then performed into the distal vasculature, after which a flow diverter stent was placed to cover the aneurysm adequately. The device size was chosen at the discretion of the treating physician. The diameter of the stent was oversized with respect to the proximal landing zone selected in the ICA by at least 0.5 mm to achieve adequate wall apposition through the self-expanding nature of the device.

### Data analysis

Data was gathered regarding patient demographics, aneurysm characteristics, and the geometry of the target vessel in which the flow diverter stent was placed. Information regarding the specifics of each stent used was collected and verified to adequately assess the degree of oversizing when the stent diameter was chosen. Assessment of the vessels and treatment effects was based on intraoperative digital subtraction angiography (DSA), intraprocedural three-dimensional (3D)-rotational angiography, and control DSA and MRI.

**Figure 1:**
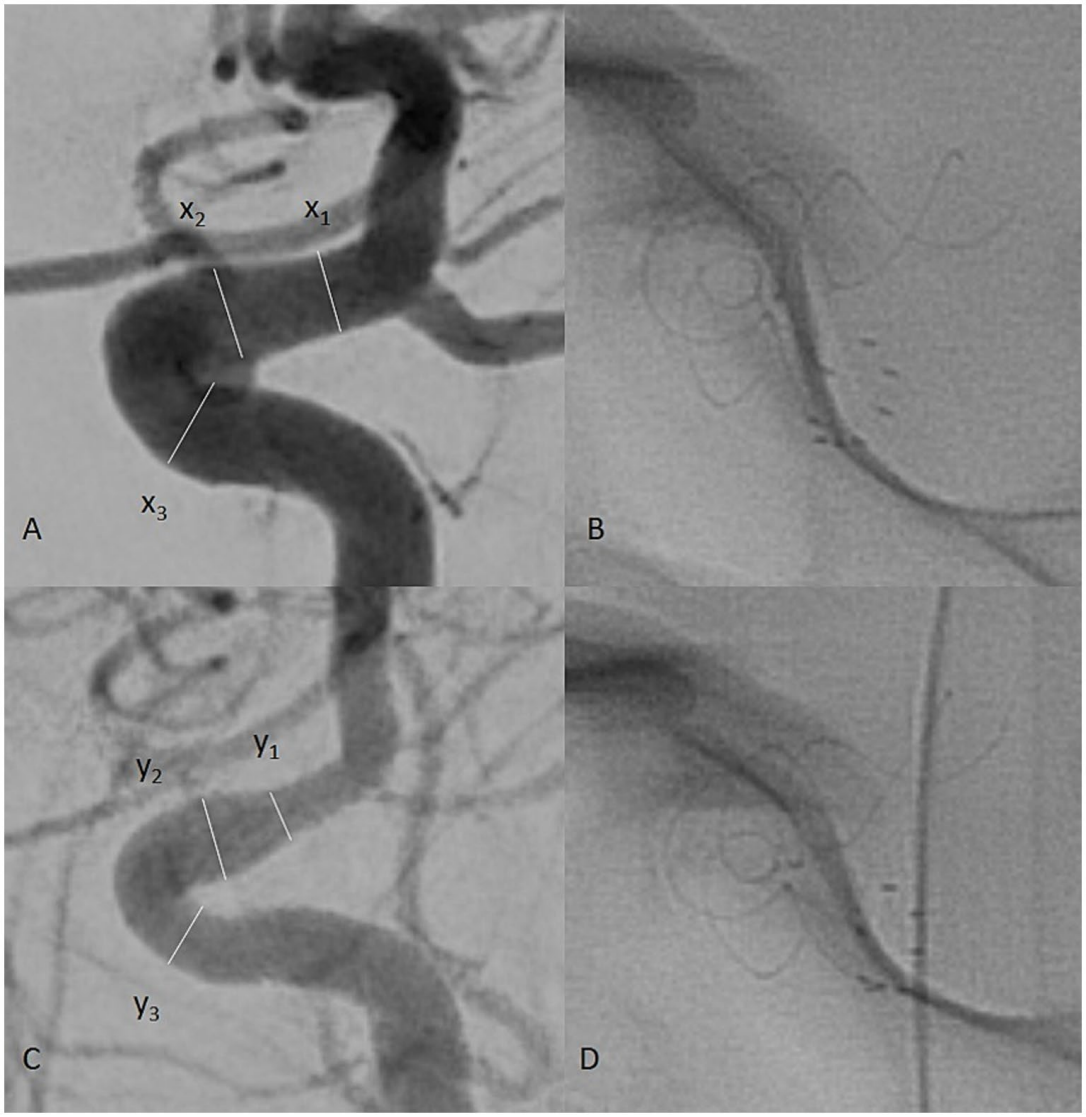
Intraprocedural digital subtraction angiography (DSA) of a patient with a left internal carotid artery (ICA) aneurysm **(A)**. The measured vessel diameters are labeled x_1_, x_2_, and x_3_ for the distal, middle, and proximal landing zones, respectively. On mid-term follow-up, another DSA was performed **(C)**. The vessel was measured at the same points of the carotid artery, labeled y_1_, y_2_, and y_3_. The formula (*x* − *y*/*x*)% was used to calculate the vessel diameter reduction as a percentage, representing the observed ISS. Single-shot images of the implant during the procedure **(B)** and follow-up **(D)** were used to assess its state.

The presence of in-stent stenosis was interpreted according to the narrowing of the parent vessel lumen diameter by comparing the intraoperative and control images. Vessel diameters were measured at three points: the proximal landing zone of the implant, the middle zone, and the distal landing zone. The formula used to determine the ISS percentage was (*x* − *y*/*x*)%, where *x* is the preoperative segmental lumen diameter, and *y* is the postoperative segmental lumen diameter of the artery (Figure 1).

Aneurysm location was categorized with the Bouthillier classification (24), which labels the beginning of the C6 segment at the level of the distal dural ring and the end just proximal to the origin of the posterior communicating artery. In this classification, the C7 segment begins at the origin of the posterior communicating artery and ends at the ICA bifurcation.

### Statistical analysis

The data were analyzed in Statistical Package for the Social Sciences (SPSS) version 29.0. The statistical methods included descriptive statistics, correlation analysis, and multiple linear regression analysis. Kolmogorov–Smirnov and Shapiro–Wilk tests were used to test the normality of the data distribution. The accepted significance level was *α* = 0.05, defined as *p* < 0.05.

## Results

### Patient demographics

A total of 137 patients treated between September 2022 and May 2023 met the inclusion criteria and were enrolled in the study. The mean patient age was 51.8 years (standard deviation [SD] ± 11.3); 86.1% were women (*N* = 118); 19% were active smokers; and 54% had a history of hypertension. The pretreatment Modified Rankin Scale (mRS) scores were 0 in 43.8%, 1 in 39.4%, and 2 in 16.8% of patients. The average diameters of the proximal, middle, and distal landing zones of the ICAs in which the stents were placed were 4.14 mm (SD ± 0.46), 3.90 mm (SD ± 0.45), and 3.61 mm (SD ± 0.45), respectively.

### Aneurysm characteristics

A total of 142 aneurysms were treated with flow diversion: 76.76% (*N* = 109) located at the ophthalmic (C6) segment of the ICA and 23.24% (*N* = 33) situated in the terminal (C7) segment of the ICA. In five patients, two aneurysms were treated with a single implant, which covered both aneurysmal necks. The mean geometric characteristics of the aneurysms were as follows: height, 5.3 mm (SD ± 3.6); neck, 3.4 mm (SD ± 2.2); and dome width, 5.5 mm (SD ± 3.8). The mean dome-to-neck ratio was 1.39 (SD ± 0.57), and the mean aspect ratio was 1.38 (SD ± 0.69).

### Stent characteristics

A total of 137 stents were placed during all procedures. The only stent type used during this study was the first-generation p64 flow diverter stent (Phenox, Germany), which was delivered in all cases *via* a Phenom 27 microcatheter. The smallest diameter implant was 3 mm, and the largest was 5 mm. The most frequently used implant diameter was 4.5 mm (in 50.36% of cases), followed by 5 mm (in 27.01% of cases; Table 1). Only one implant with a diameter of 3 mm and one with a diameter of 3.5 mm were used. The mean oversizing was 0.36 mm (SD ± 0.33) in the proximal zone, 0.60 mm (SD ± 0.34) in the middle zone, and 0.89 mm (SD ± 0.41) in the distal zone. Coiling was performed in a prior procedure in 19% (26/137) of cases. In 1.5% (2/137) of cases, intraprocedural coiling was performed before stent placement.

**Table 1:** Demographic characteristics, aneurysm characteristics, stent characteristics, and data regarding the mid-term follow-up of patients.

Data characteristics of the cases	
Patient demographics (*N* = 137)	
Age (years)	51.8 (SD ± 11.3)
Sex	Female 86.1% (118/137)
Smokers	19% (26/137)
Chronic hypertension	54% (74/137)
Admission mRS scores	0, 43.8%; 1, 39.4%; 2, 16.8%
Patients with multiple aneurysms	3.65% (*N* = 5)
Aneurysm characteristics (*N* = 142)	
Location	C6, 76.76% (*N* = 109); C7, 23.24% (*N* = 33)
Height (mm)	5.3 mm (SD ± 3.6)
Neck (mm)	3.4 mm (SD ± 2.2)
Dome (mm)	5.5 mm (SD ± 3.8)
Dome-to-neck ratio	1.39 (SD ± 0.57)
Aspect ratio	1.38 (SD ± 0.69)
Stent diameter	
5 mm	27.01% (37/137)
4.5 mm	50.36% (69/137)
4 mm	21.17% (29/137)
3.5 mm	0.73% (1/137)
3 mm	0.73% (1/137)
Safety profile	
Technical complications	5.1% (seven cases)
Late complications related to ISS	1.46% (2/137)
Occlusion rates	
Adequate occlusion rates	80.28% (114/142)
Preoperative vessel diameters	
Proximal zone	4.14 mm (SD ± 0.46)
Middle zone	3.90 mm (SD ± 0.45)
Distal zone	3.61 mm (SD ± 0.45)
Stent oversizing	
Proximal zone	0.36 mm (SD ± 0.33)
Middle zone	0.60 mm (SD ± 0.34)
Distal zone	0.89 mm (SD ± 0.41)
In-stent stenosis evaluation at follow-up	
Overall incidence	75.18% (103/137)
Proximal zone ISS values	*m* = 5% (IQR = 0, 11%)
Middle zone ISS values	*m* = 5% (IQR = 1, 13%)
Distal zone ISS values	*m* = 11% (IQR = 4, 26%)

IQR, interquartile range, ISS, in-stent stenosis; *m*, median; *N*, number; SD, standard deviation.

### Safety evaluation

**Figure 2:**
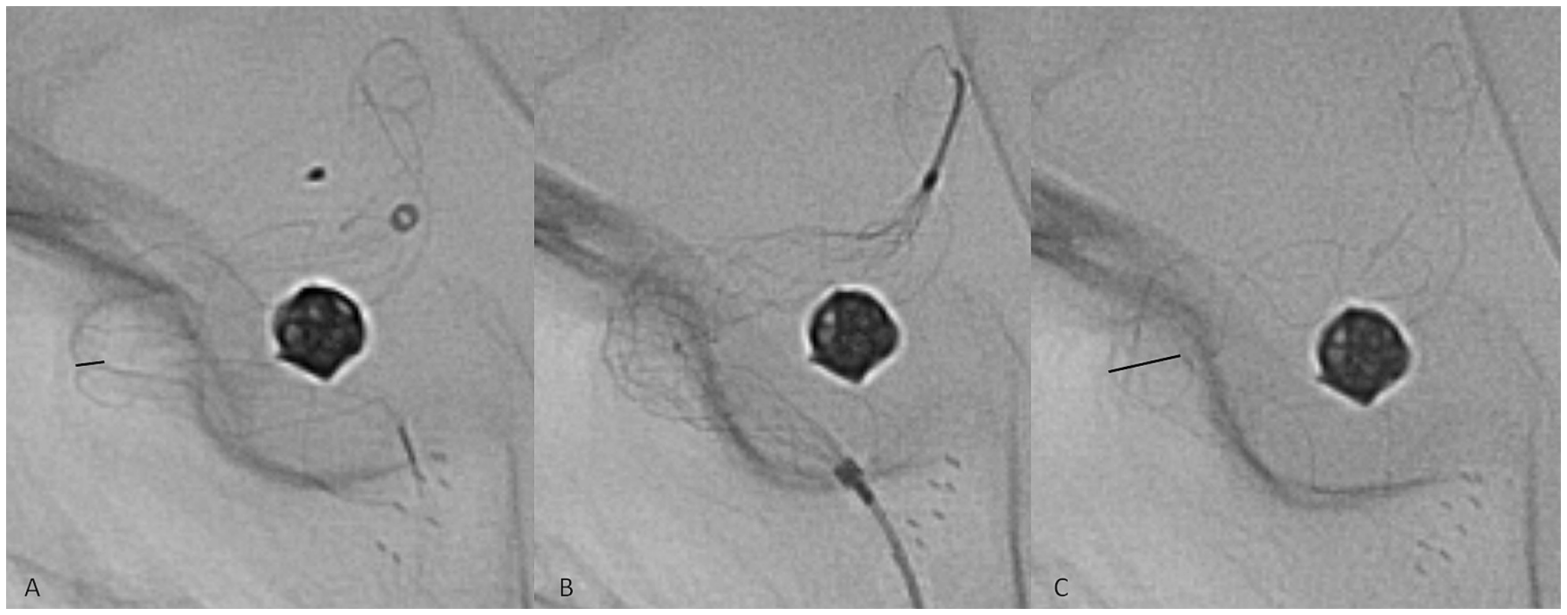
Representative case of a technical complication during flow diverter stent delivery. The twisting of the implant in the middle zone of a curve was observed (**A**, black line, representing the diameter of the twisted segment of the implant). A Comaneci temporary stent **(B)** was passed through the Phenom 27 microcatheter, and the expansion of the Comaneci stent inside the twist opened the p64 completely (**C**, black line).

No immediate peri- or postprocedural clinical complications associated with therapy were observed. The technical complication rate associated with device delivery was 5.1%: in seven cases, implant deformation during the procedure was observed, with inadequate wall apposition. In all of these cases, angioplasty was performed to remedy the issue with a Comaneci temporary stent (Rapid Medical, Israel), which was passed through the 0.027″ stent delivery microcatheter (Figure 2). The temporary stent was opened at the deformation site until satisfactory results were achieved. No thromboembolic or hemorrhagic complications associated with the angioplasty were observed. Late clinical manifestations were observed several days after prasugrel discontinuation at the 1-year follow-up in two patients with ISS. The patients reported contralateral weakness of the limbs, and one of them had accompanying contralateral facial palsy and transitory aphasia. DAPT was again prescribed, and the symptoms spontaneously resolved in both patients within several days.

### Follow-up and assessment of parent vessel in-stent stenosis

After discharge from the hospital, all patients were scheduled for follow-up angiography/MRI and prescribed DAPT consisting of 10 mg prasugrel and 100 mg aspirin once daily until then. The average follow-up time was 13.2 months (SD ± 2.2). Digital subtraction angiography was performed in 88.2% of cases, whereas, because of patient refusal to undergo DSA, an MRI with silent time-of-flight sequences was conducted in 11.8% of cases. An adequate aneurysm occlusion rate (complete exclusion from the intracranial circulation or neck remnant only) was observed in 80.28% of cases. After the follow-up, the same segments of the ICA as those during the procedure were measured. The average diameters of the proximal, middle, and distal landing zones at follow-up were 3.83 mm (SD ± 0.56), 3.58 mm (SD ± 0.56), and 3.03 mm (SD ± 0.71), respectively. Any decrease in vessel diameter in the segments of the stented artery at follow-up was defined as ISS. According to those criteria, the overall incidence of ISS was 75.18% (103/137). The mean percentage values of ISS were 7.2% proximally, 8.6% in the middle zone, and 16.1% in the distal zone. The respective median values were 5, 5, and 11%, respectively (Table 1). The single most significant ISS was observed in the distal landing zone and showed approximately 70% lumen reduction at follow-up; therefore, the data contained substantial outliers, which must be considered when interpreting the findings.

A multiple regression analysis was performed to assess the effects of risk factors such as sex, age, smoking status, and history of hypertension on the ISS observed in the cohort.

The independent variables significantly predicted ISS after flow diversion, *F*(4, 406) = 12.428, *p* < 0.001, thus indicating that the four factors overall were associated considerably with ISS development. The value of *R^2^* = 0.109 suggested that the model accounted for 10.9% of the variance in the dependent variable.

Additionally, coefficients were assessed to ascertain the influence of each factor on the independent variable. Smoking (*B* = −0.037, *t* = −2.308, *p =* 0.021) and older age (*B* = −0.030, *t* = −5.661, *p* < 0.001) were negatively associated with ISS development. Sex and hypertension had no statistically significant predictive values for ISS development. This analysis suggested that smokers and older patients had a lower chance of developing substantial ISS but accounted for a small amount of the overall variance.

A correlation analysis assessed the effects of stent oversizing on ISS. Considering the oversizing and vessel diameters in the various measured segments of the ICA, a slight statistically significant correlation was found between stent oversizing and ISS development (*r*: 0.099, *p* = 0.022). When only the distal landing zone was evaluated, the strength of this correlation increased significantly (*r*: 0.210, *p* = 0.014). These findings indicated that ISS risk increases disproportionately with greater stent oversizing, as occurred in the distal landing zones.

## Discussion

The formation and clinical significance of ISS remain unclear. Studies on flow diversion have indicated markedly different rates of this complication, from 8% to more than 40% (13, 16, 25). ISS is frequently reported to have a benign clinical course and has been observed to regress in follow-up in some studies spontaneously (26). The presented clinical experience in this study indicated a much higher percentage than previously reported, because of the narrow threshold for defining any vessel reduction as ISS. Despite the definition used, only two of the patients in the study exhibited any notable chronic clinical symptoms during follow-up, which were attributed to the observed stenosis. Thus, ISS can be considered a mostly “quiet” complication that theoretically can spontaneously resolve. However, in severe cases of ISS, the prolonged time during which it forms also enables the dilation of collateral pathways supplying regions of the brain that develop vascular deficiency (25). To date, the management of ISS by treating physicians has mainly been conservative, with few to no options for acute management (27). DAPT is frequently prolonged until subsequent follow-up. In the described cases of symptomatic ISS, that strategy was also adopted and achieved satisfactory results. Some authors have indicated that angioplasty is an invasive technique that can resolve stenosis; however, its inherent risks, such as parent vessel damage and embolic complications, must be weighed against its potential benefits (16). Another potential option for dealing with this phenomenon has already been implemented in cardiology, with the prescription of Cilostazol for patients with coronary stent restenosis (28). This drug’s effect on intimal overgrowth can be linked with suppression of P-selectin expression on platelets and the following upregulation of leukocyte Mac-1 (29). Some authors have already published their evaluation of the efficacy and safety of Cilostazol in patients with ISS after flow diversion with promising results (30). Although ISS is rarely symptomatic, its complications can be dire and challenging to address; therefore, preventive strategies are the most logical course of action. To achieve prevention, identification, and avoidance of risk factors associated with ISS development would provide the most benefits for patients. Narrowing the therapeutic range of flow diverters to primarily elective cases might yield satisfactory results.

The study included only patients with supraclinoid aneurysms of the ICA for several reasons. First, these segments of the cerebral vasculature are relatively easy to assess on both DSA and MRI, and they could be reliably measured in different projections. Second, aneurysms at the ophthalmic and terminal segments are among the most common locations of this pathology; therefore, a substantial number of patients could be included to interpret their risk factors in the context of ISS. Finally, their use in treating smaller aneurysms markedly increased after the initial introduction of flow diverters for treating wide-neck bifurcation aneurysms. Flow diversion has become a mainstream treatment for aneurysms at this location, because of its high technical success rate and favorable safety profile (31). Therefore, a specific evaluation of the risk factors and clinical significance of ISS in the distal ICA might most reliably benefit patients undergoing endovascular treatment of cerebral aneurysms at that location. The findings could be implemented in the context of flow diversion in more distal vascular territories, such as the middle and anterior cerebral arteries (32).

Several factors might potentially be responsible for ISS development. One of the most crucial and bioactive components of the vessel wall is the endothelium, which is involved in the early response to hemodynamic changes to maintain vessel wall homeostasis. Endothelial cells have mechano-transductor properties, which are implemented through their intra- and extracellular matrix components, and stretch-activated ion channels (33, 34). Any changes in laminar flow or wall shear stress induce cell reactions in the cells to maintain normal blood flow’s physiological conditions. When a stent is delivered, several pathological processes occur, including endothelial damage, inflammatory reactions, and microflow disturbances near the struts of the stent (35). The implanted devices exert mechanical stress, because of their self-expanding nature. With oversizing, the stent maintains a constant radial force, aiming to reach the device’s nominal diameter. Consequently, the stent is comparable to a “loaded spring” that continually applies outward pressure on the vessel, which further increases with greater oversizing (36, 37). The mechanical damage to endothelial cells caused by device implantation also hinders their ability to produce vasoactive substances, such as nitrous oxide, which regulates thrombus formation and cell proliferation (19). All of these conditions are a prerequisite for endothelial dysfunction, pathological reparation, and aggressive neo-proliferation to occur. In this sample of patients, it was observed that the distal landing zone of the stents was more prone to ISS formation. Possibly, the pathological process in that segment might be exacerbated with greater oversizing. A mechanical threshold for the vessel wall might exist, beyond which a more pronounced reaction is induced. Oversizing considering not only the proximal landing zone but also the distal landing zone might remedy some ISS cases, according to the statistical analysis.

Cell senescence is another crucial protective factor against ISS. Studies have indicated that younger age is an independent risk factor for ISS formation (12, 38). Diminished cell proliferation ability in older patients has been suggested as the reason for the lower incidence and severity of ISS in this population. This series is consistent with this explanation, as lower rates of ISS in older patients were evaluated in the study.

The evaluation of the effects of sex on ISS did not reach statistical significance, possibly because the study included substantially more women than men, thus potentially resulting in bias. Nevertheless, other studies have demonstrated that women have an elevated risk of developing significant ISS (12, 38).

A perhaps more controversial result from the analysis pertained to the effects of smoking on ISS. Smokers in the study developed less significant stenosis than non-smokers at follow-up. Smoking increases the overall cardiovascular risk of every procedure, because of its effects on innate thrombocyte reactivity, vessel rigidity, and enhancing anatomical tortuosity. Its relative protective value can be attributed to its contribution to chronic vessel damage and endothelial dysfunction (39), through which it also raises the presumed threshold of iatrogenic damage necessary for ISS to become pronounced.

In summary, stent oversizing is a crucial condition for successfully treating cerebral aneurysms with flow diversion. According to the manufacturer’s recommendations, the devices were oversized with respect to the most significant part of the vessel to be stented. The use of a stent diameter greater than the vessel diameter is aimed at achieving several goals. First, the stent achieves adequate wall apposition because of the constant application of outward radial force, thereby decreasing the risk of acute thrombosis between the stent struts and the uncovered endothelial layer. Another benefit of oversizing is diminished risk of aneurysmal endoleak, which might compromise treatment outcomes (40). A key aspect of oversizing is the decreased risk of stent migration Finally, oversizing increases the implant porosity, which might aid in keeping the covered side branches patent and functional (41). A drawback of oversizing observed herein was that this increased porosity was a risk factor for aneurysm occlusion, because of the diminished flow disturbance inside the sac. Thus, oversizing is a core technical step in flow diversion therapy, but, to date, no attention has been paid to the other end of the spectrum. The results from this study emphasize the need for more precise recommendations regarding the correct choice of stent size, considering all arterial segments.

## Limitations

One main limitation of this study is that all patients were treated with a single device type. Additional studies assessing other flow diverter stents are needed to appreciate stent oversizing as a risk factor for ISS fully. Another potential limitation is that not all patients were followed up through DSA, thus potentially introducing bias in the target vessel measurement results.

## Conclusion

ISS is observed in many patients treated with flow diversion. Most frequently, this phenomenon is completely asymptomatic, and symptomatic cases can often be managed through conservative means, such as DAPT continuation. This study observed a significant correlation between the degree of stent oversizing and ISS incidence and severity at mid-term follow-up. Based on these findings, it may be recommended to use device diameters accounting for both the proximal and distal landing zones of the target vessel. Older patients and smokers had less severe ISS than other patients in the cohort at follow-up.

## Data Availability

The raw data supporting the conclusions of this article will be made available by the authors, without undue reservation.
